# Pacifier Use and Its Influence on Pediatric Malocclusion: A Scoping Review of Emerging Evidence and Developmental Impacts

**DOI:** 10.3390/dj13070319

**Published:** 2025-07-14

**Authors:** Man Hung, Jacob Marx, Corban Ward, Connor Schwartz

**Affiliations:** 1 College of Dental Medicine, Roseman University of Health Sciences, South Jordan, UT 84095, USA; 2 Division of Public Health, University of Utah, Salt Lake City, UT 84108, USA; 3 College of Education, University of Utah, Salt Lake City, UT 84112, USA; 4 Library, Roseman University of Health Sciences, South Jordan, UT 84095, USA; 5 Library, Noorda College of Osteopathic Medicine, Provo, UT 84606, USA

**Keywords:** child, preschool, dental development, malocclusion, oral health, pacifiers

## Abstract

**Background/Objectives**: Pacifier use is a widespread soothing practice during infancy, but extended use has been linked to adverse dental outcomes, particularly malocclusion. This review aimed to evaluate the association between pacifier use and dental developmental issues in infants and toddlers and to identify key influencing factors. **Methods**: A scoping review using PubMed, Scopus, Web of Science, and Dentistry and Oral Sciences Source was performed. Peer-reviewed, full-text articles published in English between 2014 and 2024 were screened by two independent reviewers using predefined criteria. Eligible studies were thematically analyzed. **Results**: From 262 records, 35 studies met the inclusion criteria. Pacifier use was consistently associated with an increased prevalence of malocclusions, including anterior open bite, posterior crossbite, and overjet. The risk and severity of dental issues were strongly influenced by the duration, frequency, and intensity of pacifier use. Prolonged use beyond three years significantly increased the likelihood of structural changes requiring intervention. **Conclusion**: There is strong evidence linking pacifier use to negative dental developmental outcomes, particularly when use is prolonged or frequent. Early intervention, caregiver education, and timely weaning are critical to minimizing long-term oral health risks. Future research should explore pacifier design, objective measures of use, and how socioeconomic factors may influence pacifier use patterns and oral health outcomes. Understanding these relationships can support the development of more targeted and equitable prevention strategies.

## 1. Introduction

The infant and toddler years are critical for oral and dental development, as they encompass several key milestones essential for healthy oral function [1]. During this early period, infants develop a sucking reflex, experience teething, establish primary occlusion, and undergo significant growth of oral structures—particularly the mandible and palate [2,3]. Dental development specifically refers to the formation, eruption, and alignment of primary teeth, which occur in tandem with broader oral cavity changes. Oral cavity development is influenced by both biological processes and environmental interactions, including caregiving practices [4]. Proper care during this formative stage lays the foundation for future health, well-being, and developmental outcomes.

Among common caregiving practices, pacifier use has become a widespread method for soothing infants and toddlers [5]. Pacifiers allow infants to engage their natural sucking reflex between feedings and can also provide relief during teething [6]. Interestingly, even parents who initially intend to avoid pacifiers may adopt their use as a practical solution for settling their infants [7]. In addition to soothing benefits, pacifier use has been associated with a reduced risk of sudden infant death syndrome [8].

However, despite their immediate advantages, pacifier use has raised concerns regarding its long-term impact on dental health [9]. Prolonged use is associated with various dental complications, most notably malocclusions, including anterior open bite, posterior crossbite, and increased overjet [10,11]. Improper alignment can lead to difficulties in swallowing, speech development, and an increased susceptibility to dental trauma [12]. Additionally, pacifier use may interfere with the normal growth of the oral cavity, affecting tooth positioning and palate formation [13,14]. Bruxism has also been reported as a possible outcome of extended pacifier use [15]. These issues often necessitate orthodontic treatment, underscoring the importance of evaluating pacifier-related risks early.

Given the potential long-term implications, it is important to assess pacifier use within the broader context of infant and toddler care and to explore strategies that minimize negative outcomes. While numerous clinical trials and surveys have addressed this topic, there remains a lack of up-to-date comprehensive reviews synthesizing recent findings. Although previous reviews exist [16,17], none have incorporated the growing body of research from the last several years, published after 2018.

Recent systematic reviews have explored pacifier use in the context of breastfeeding [18,19], offering valuable insights, but these findings may not be applicable to families who are unable to breastfeed. Similarly, broader reviews of non-nutritive sucking habits [20,21] provide useful context, but pacifier habits are generally more modifiable than behaviors like digit sucking. By focusing specifically on pacifier use, this review aimed to provide practical, evidence-based guidance that can be more readily applied in clinical and caregiving settings.

Understanding the balance between the soothing benefits of pacifier use and its potential dental risks is essential for making informed decisions that promote both the immediate comfort and long-term oral health of young children [22]. This scoping review aimed to provide meaningful insights for dental professionals, parents, and caregivers seeking to optimize oral health during early childhood. Specifically, the review examined the effects of pacifier use on dental development in infants and toddlers, with a focus on potential outcomes such as malocclusions and altered oral cavity growth.

## 2. Methods

This scoping review included peer-reviewed, full-text research articles published in English between 2014 and 2024 that examined the relationship between pacifier use and dental developmental complications. The study was conducted following the Preferred Reporting Items for Systematic Reviews and Meta-Analyses and extension for Scoping Reviews (PRISMA-ScR) Guidelines [23]. Eligible studies specifically investigated outcomes related to tooth positioning, oral cavity growth, or other dental structures in infants and toddlers. Reviews (including literature reviews, systematic reviews, and meta-analyses), editorials, letters to the editor, commentaries, and conference abstracts were excluded. Additionally, studies published more than ten years ago, those not written in English, or those that did not focus directly on pacifier use and dental outcomes were omitted to maintain the review’s relevance and specificity.

A comprehensive literature search was conducted across four major databases, namely PubMed, Scopus, Web of Science, and Dentistry and Oral Sciences Source, to assess the impact of pacifier use on children’s dental development. The search strategy employed a combination of Medical Subject Headings (MeSH) and keywords drawn from titles and abstracts. Boolean operators (AND, OR, NOT) were used to refine results. The search terms (detailed in Table 1) included pacifiers, adverse effects, odontogenesis, tooth development, dental arch, growth and development, maxilla/growth and development, maxillofacial development, oral development, and malocclusion. EndNote 21 software was used to organize the citations and automatically remove duplicate records.

Two independent reviewers (J.M. and C.W.) screened all titles and abstracts using predefined inclusion and exclusion criteria. Discrepancies were resolved through discussion or by consultation with a third reviewer (M.H.). Data extraction was conducted using a standardized form to ensure consistency across studies. Two reviewers (J.M. and C.W.) independently extracted key study details, including authors, year of publication, study design, population characteristics, objectives, interventions, measured outcomes, key findings, and conclusions. Disagreements were resolved by consensus or adjudicated by a third reviewer (M.H.). Additionally, the third reviewer (M.H.) randomly selected articles to review and verified the work of the two independent reviewers. This multi-step review process was implemented to enhance the rigor, transparency, and reliability of the findings.

## 3. Results

**Figure 1:**
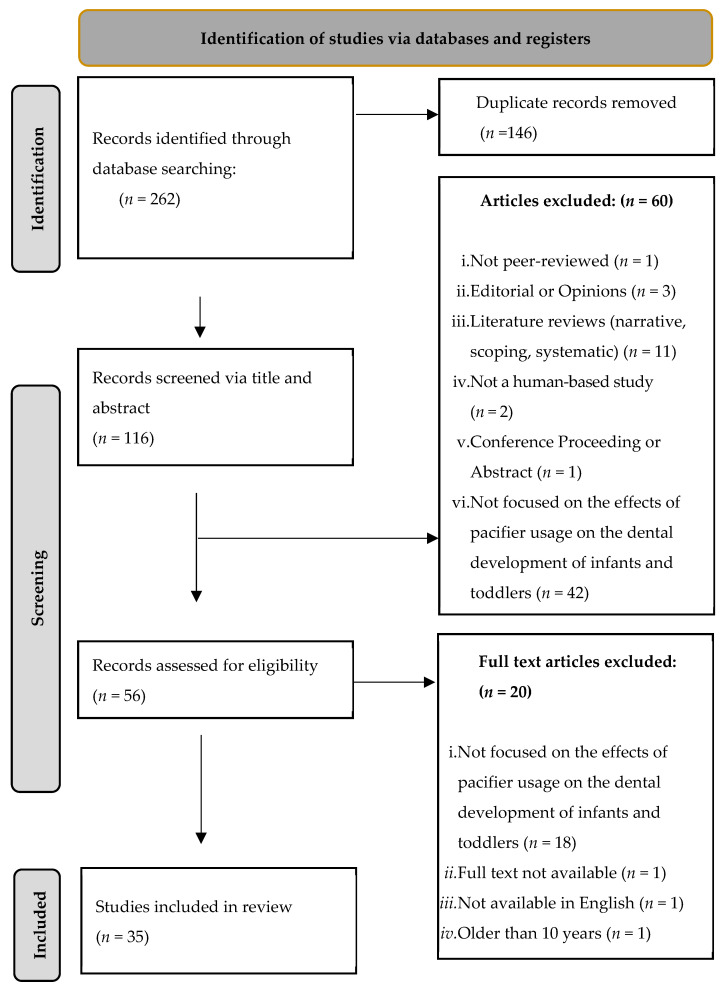
Flow diagram of article selection adapted from the PRISMA-ScR guidelines.

The database search identified 262 unique articles. After removing 146 duplicates, two independent reviewers screened the remaining 116 articles by reviewing titles and abstracts based on the predefined inclusion and exclusion criteria (outlined in Table 2). Following the initial screening, 20 additional articles were excluded due to irrelevance, a lack of full-text availability, language limitations, or publication dates exceeding 10 years. Ultimately, 35 studies met the inclusion criteria and were included in the final review (Figure 1).

The findings from these studies provide insights into the relationship between pacifier use and dental malocclusions. The results are organized into three main themes: malocclusion patterns associated with pacifier use, influential factors in pacifier use, and age-related trends (outlines in Table 3).

### 3.1. Malocclusion Patterns Associated with Pacifier Use

Pacifier use has been consistently linked to several types of malocclusion, with AOB, posterior crossbite, and increased overjet being the most frequently reported. The relationship appears to be influenced not only by the presence of pacifier use but also by its duration and frequency.

Among the most prominent findings is the strong association between pacifier use and anterior open bite [48]. Several studies reported a significantly higher prevalence of AOB in pacifier users compared to non-users. For instance, Alves et al. found that 55% of pacifier users exhibited AOB versus 14% of non-users [25]. Likewise, Traebert et al. found that 49.4% of pacifier users exhibited a statistically higher prevalence of AOB when compared to those who did not (*p* = 0.003) [55]. Al Duliamy et al. and Sousa reported an even higher prevalence of 87% [24,36], while both Lira et al. and Oliveira identified a statistically significant increase in AOB risk (*p* < 0.05) [35,45]. Notably, Oliveira reported that pacifier users were 1.83 times more likely to develop an anterior open bite [45]. Interestingly, one study found that open bite decreased significantly after children stopped using pacifiers; before pacifier removal, 85% of children had an open bite, but this dropped to just 4% one year later [53].

Posterior crossbite and overjet were other frequently observed outcomes. Miotto et al. found a 1.77-fold increased risk of crossbite in pacifier users [34], and Germa et al. noted a 36% occurrence in children who continued pacifier use beyond age three [41]. Similarly, prolonged pacifier use was linked to increased overjet. Feldens et al. and Moimaz et al. reported prevalence rates of 51.4% and 22.8%, respectively, in children using pacifiers after 12 months [38,47].

Beyond individual conditions, general occlusal health was also adversely impacted [39]. Costa et al. observed worse overall occlusal outcomes in pacifier users [31]. Wagner et al. reported that 50.5% of pacifier users developed some form of malocclusion [57], while Amaral et al. found that 62.3% of pacifier-using children had malocclusions, compared to significantly lower rates in non-users [26]. Additionally, Pegoraro et al. reported that 86.1% of pacifier users experienced malocclusion [33]. Traebert et al. found that children who used pacifiers were more likely to have a Class II or III bite, and this connection remained significant even after considering other factors (*p* < 0.05) [56]. Besides general malocclusion, distoclussion and maxillary arch growth were attributed to general occlusal health. Feldens et al. found distoclusion to be common, especially in children who used pacifiers, with 34.5% of past users and 51.7% of current users having it [37]. Zen et al. found that pacifiers significantly impact the growth of the upper jaw: children who used pacifiers experienced more growth in this area and the relationship was statistically significant (*p* < 0.05) [14].

Nonetheless, some studies reported lower prevalence rates. For example, Gomes et al. reported AOB in only 23.2% of pacifier users [43], and Moimaz et al. documented relatively low rates of 12.9% for AOB and 0.7% for posterior crossbite in children under 30 months [47]. Herrera et al. found that only 13.4% of pacifier users developed posterior crossbite [44]. Chen et al. reported that only 10% of pacifier users had excessive overjet [30]. The lowest prevalence of general malocclusion was found to be 4.9% by Rai et al. [51].

### 3.2. Influential Factors on Pacifier Use

While not all studies identified specific contributing factors, the majority consistently pointed to three primary influences on dental development: duration, frequency, and intensity of pacifier use [27,31,46]. Among these, intensity was the most difficult to measure due to its reliance on subjective parental reporting. Lima acknowledged this limitation in methodology, emphasizing the challenge of accurately quantifying sucking force or muscle engagement through survey data [46]. Despite this, Samohyl reported that even without precise measurement tools, active and forceful sucking—characterized by the engagement of stronger muscle forces—was consistently associated with adverse effects on oral development [52]. Muscular forces—including tongue positioning and thrusting—were also mentioned by Duliamy as large contributing factors to mandibular width and AOB, respectively [24].

Frequency of use also demonstrated a notable impact. Several studies found a positive correlation between how often a pacifier was used and the severity of dental complications. For instance, Alves and Costa both noted that a higher usage frequency increased the risk of malocclusion [25,31]. Nihi et al. quantified this relationship, reporting a prevalence ratio of 11.33 for anterior open bite with increased pacifier frequency, suggesting a strong dose–response relationship [49].

However, duration emerged as the most influential factor across studies. Lima identified the length of pacifier use as the strongest predictor of various malocclusion types, surpassing both frequency and intensity in significance [46]. Supporting this, Assis et al. found that duration alone accounted for 28.2% of the variation from normal dental development [28]. Silvestrini-Biavati et al. showed that 78.3% of children with AOB were still using a pacifier, and all children who continued pacifier use at age 5 had AOB [54]. This highlights the importance of early intervention and limiting long-term pacifier use to reduce the risk of long-lasting oral health issues.

### 3.3. Age-Related Trends

Several studies demonstrated a clear association between shorter durations of pacifier use and a reduced incidence of dental abnormalities. For instance, Golovachova reported that continued use beyond 18 months was associated with an increase in the prevalence of AOB from 9.3% to 13.8%, and an increase in Class II malocclusion from 22.5% to 27.9% [42].

A commonly identified critical threshold across studies was the age of three years. Numerous findings indicated that the prevalence of both anterior open bite and posterior crossbite increased significantly when pacifier use extended beyond this age. Cardoso et al. observed that children under the age of three who used pacifiers exhibited an AOB prevalence of 18.8%, while those who continued use past three years showed a markedly higher prevalence of 65.1% [29]. Similarly, Galan-Gonzalez et al. found that the AOB prevalence increased from 22.3% in children using pacifiers before the age of three to 35.5% in those who continued beyond that age [40]. Pimenta et al. observed that pacifier use after the age of three was more often linked to distocclusion (*p* = 0.01) and open bite (*p* < 0.05) [50].

Additional evidence reinforces the impact of prolonged pacifier use on the risk of malocclusion. Da Rosa reported that pacifier use up to 48 months elevated the likelihood of malocclusion by approximately 5- to 15-fold [32]. Likewise, Assis et al. found that pacifier use extending beyond 18 months increased the risk of anterior open bite by a factor of 3.2 [28].

## 4. Discussion

This scoping review highlights a strong and consistent association between pacifier use and various patterns of malocclusion in children. The evidence across studies indicates that pacifier use is linked to an increased prevalence of AOB, posterior crossbite, increased overjet, and general occlusal misalignment. Importantly, three key behavioral factors, namely duration, frequency, and intensity of pacifier use, were identified as critical factors influencing the severity and persistence of malocclusion. Among these, prolonged use consistently emerged as the most significant, with studies uniformly reporting higher rates of malocclusion when pacifier use extended beyond early childhood.

Although a minority of studies reported lower prevalence rates of malocclusion among pacifier users, the overall body of evidence underscores the detrimental impact of pacifier habits on dental development. This reinforces the need for early intervention and targeted parental education to minimize long-term consequences on children’s oral health.

The prevalence of AOB was the most notable in our findings; this aligns with previous research on non-nutritive sucking habits. Sadoun et al. reported that children with habitual non-nutritive sucking behaviors—such as pacifier or digit use—were significantly more likely to develop AOB [20]. Likewise, multiple studies noted that posterior crossbite, though less prevalent than AOB, was less closely linked to pacifier habits. Arpalahti and Melink et al. both emphasized that a longer duration of pacifier use was a key risk factor in the development of crossbite [27,58].

Duration and frequency were consistently associated with the degree of oral and occlusal alteration. Studies such as those by Nihi, Warren, and Schmid all found that longer and more frequent pacifier use correlated with increased malocclusion severity [17,49,59]. However, measuring intensity remains a methodological challenge, as it is typically reliant on subjective parental reporting. Nevertheless, when intensity was evaluated—such as in the study by Samohyl—forceful sucking was also linked to negative developmental outcomes [52].

Age was also found to be a critical variable. Children who discontinued pacifier use before the age of three showed substantially lower rates of malocclusion. This finding aligns with the guidelines of the American Academy of Pediatric Dentistry [60], which recommends that children discontinue pacifier use by the age of three. Furthermore, studies such as those by Poyak and Christensen have shown that some occlusal changes may self-correct if pacifier use is discontinued early, supporting the importance of timely weaning [61,62].

### 4.1. Impact

When malocclusions persist beyond early childhood, orthodontic treatment becomes the standard corrective approach. This may include braces, clear aligners, or other devices designed to reposition teeth and improve occlusal function [63]. Research by Peres indicates that misalignment that develops during the primary dentition stage significantly increases the likelihood of needing orthodontic care in adolescence [64]. While such interventions can be effective, they are often costly and may be inaccessible to families facing socioeconomic barriers. In this context, the findings of this review are especially relevant: by addressing modifiable behaviors early—such as pacifier use—families can potentially avoid the need for extensive and expensive treatments later in life.

Furthermore, the impacts of malocclusion go beyond functional concerns. Masood et al. emphasized the negative influence of poor occlusion on oral health-related quality of life [65]. Children may experience difficulties in speaking, eating, and even facial discomfort or pain. Aesthetic concerns related to misaligned teeth can also contribute to reduced self-confidence, especially during school-age years [66].

Speech impairments are another consequence of misaligned dentition. Proper articulation requires the correct positioning of the tongue, teeth, lips, and airflow—all of which can be disrupted by malocclusion [67,68]. When left unaddressed, these issues may hinder communication, academic performance, and self-esteem [69]. Understanding the role of pacifier habits in early orofacial development is therefore essential not only for dental health but also for speech and language development [70].

Chewing difficulties also result from occlusal misalignment, as noted by Magalhães [71]. While not typically associated with severe nutritional deficits, this can limit dietary variety and pose challenges in maintaining a balanced, healthy diet. Over time, these difficulties may further compound a child’s health outcomes. Although children often adapt to such challenges, psychosocial consequences become more pronounced with age. As children become more aware of their appearance, dental irregularities can impact their self-image and social behavior. Research has shown that developmental differences in dental and facial appearance may lead to social withdrawal, a reluctance to smile or speak, and diminished self-esteem [72,73,74]. These psychological outcomes highlight the importance of preventive measures aimed at reducing non-nutritive sucking habits during early childhood.

A study by Degan et al. found that professionally guided interventions explaining health risks were the most effective for cessation, with a 90% success rate. Other methods like applying unpleasant substances (80%) and sudden removal (64%) were also effective, while parental explanation alone had limited success (38%) [75]. These findings suggest that clear, structured guidance provided by health professionals can significantly support caregivers in helping children wean from pacifiers. A controlled 12-month clinical trial in 4-year-old children showed that interrupting pacifier use not only reversed malocclusion, but also improved breathing and speech functions, effectively correcting the oro-dentofacial changes caused by the habit [53]. For dental practitioners, this underscores the importance of communicating to parents that stopping pacifier use is not just preventative; it can actively restore arch development and oral function. Clinicians should use clear, positive messaging supported by visual aids, set structured weaning plans, and maintain regular follow-up to reinforce progress and motivate lasting habit change.

### 4.2. Limitations

While this review provides a comprehensive synthesis of current literature on pacifier use and dental development, several limitations should be acknowledged. First, although we conducted a robust search across major databases, the number of databases included was limited, which may have led to the omission of some relevant studies. Similarly, we restricted inclusion to English-language publications, which may have excluded valuable research published in other languages. These decisions were made to ensure feasibility and consistency in interpretation but may impact the overall breadth of the review.

Another limitation was the broad age range of study participants across the included articles. While this approach allowed for a more inclusive review of the literature, it may have diluted the age-specific effects of pacifier use, particularly in critical developmental windows. Additionally, we included studies with a range of designs and outcome measures. This methodological diversity, while reflecting the current state of research, introduced variability that made direct comparisons challenging.

We also acknowledge the lack of pacifier diversity that was included in the study. There are many different types of pacifiers currently on the market—including orthodontic pacifiers, round pacifiers, and flat pacifiers. Along with different shapes, there are also different materials and constructions. Due to a lack of research involving and comparing a diversity of pacifiers, studies that focused on the differences between types of pacifiers were not included. While this limited our results, it also insured that only studies focused on comparing pacifiers and malocclusion were included, eliminating any potential bias that may have come from a comparison of pacifiers.

Despite these limitations, the strength of this review lies in its systematic approach, its emphasis on clinically relevant outcomes, and the synthesis of emerging patterns across a diverse body of literature. The findings offer valuable insights and provide a strong foundation for future targeted research and clinical recommendations.

### 4.3. Future Direction

Future research should investigate the impact of pacifier design, particularly by comparing orthodontic and conventional pacifiers, on dental and orofacial development. Currently, evidence is limited regarding whether specific designs may mitigate the risk of malocclusion. This area of inquiry can be complicated by ethical considerations—particularly the challenge of intentionally assigning infants to potentially harmful interventions—and by practical issues related to infant acceptance of unfamiliar pacifier types. Nevertheless, well-designed observational or longitudinal studies could offer meaningful insights while minimizing ethical concerns.

Further studies are needed to disaggregate the individual effects of pacifier use behaviors, specifically duration, frequency, and intensity. While these factors are frequently acknowledged in existing literature, few studies have examined them independently using objective and standardized measurements. A deeper understanding of which behaviors exert the greatest influence on oral development could lead to more nuanced and effective guidance for both parents and product designers.

In addition, socioeconomic status was identified in several studies as a potential moderator of pacifier use patterns and related outcomes. Future research should more thoroughly explore the roles of income, caregiver education, access to dental care, and health literacy in shaping pacifier habits and their long-term dental implications. Clarifying these relationships could inform targeted public health strategies aimed at supporting at-risk populations and reducing disparities in early childhood oral health. By addressing these gaps, future studies can enhance our understanding of modifiable risk factors and contribute to the development of evidence-based recommendations that are both practical and equitable.

## 5. Conclusions

This review underscores the clear link between pacifier use and dental developmental issues, particularly malocclusion. The risk of these outcomes increases with prolonged or frequent use, highlighting the importance of early intervention. By understanding and addressing modifiable factors—such as duration, frequency, and intensity—caregivers and health professionals can take proactive steps to support healthy oral development. Promoting awareness and timely weaning is essential to reducing the likelihood of long-term complications. Clinicians should have the best interest of patients in mind when providing parents with guidelines to promote lasting habit changes or prevent or reverse issues that develop from pacifiers.

## Figures and Tables

**Table 1:** Database search strategies employed.

Database (Date of Search)	Search Strategies	Number of Articles Found
PubMed (22 November 2024)	(“Pacifiers/adverse effects”[Mesh] OR “Pacifiers”[Mesh] OR “Pacifiers”[tiab] OR “Pacifier”[tiab]) AND (“Odontogenesis”[Mesh] OR “Odontogenesis” OR “Tooth Development” OR “Dental Arch/growth and development”[Mesh] OR “Dental Arch” OR “Maxilla/growth and development”[Mesh] OR “Maxillofacial Development”[Mesh] OR “Oral Development” OR “Maxillofacial Development” OR “Malocclusion”[Mesh] OR “Malocclusion”)	73
Scopus (22 November 2024)	TITLE-ABS((“Pacifiers” OR “Pacifier”) AND (“Odontogenesis” OR “Tooth Development” OR “Dental Arch” OR “Maxilla” OR “Oral Development” OR “Maxillofacial Development” OR “Malocclusion”)) AND PUBYEAR > 2013 AND PUBYEAR < 2025 AND (LIMIT-TO (LANGUAGE,”English”))	72
Web of Science (22 November 2024)	TS = ((“Pacifiers” OR “Pacifier”) AND (“Odontogenesis” OR “Tooth Development” OR “Dental Arch” OR “Maxilla” OR “Oral Development” OR “Maxillofacial Development” OR “Malocclusion”))	58
Dentistry and Oral Sciences Source (22 November 2024)	(“Pacifiers” OR “Pacifier”) AND (“Odontogenesis” OR “Tooth Development” OR “Dental Arch” OR “Maxilla” OR “Oral Development” OR “Maxillofacial Development” OR “Malocclusion”)	59

**Table 2:** Inclusion and exclusion criteria employed during the data screening.

Inclusion Criteria	Exclusion Criteria
Published peer reviewed articles in English Research articles from 2014 to 2024 Focused on the relationship between pacifier use and dental development complications caused by pacifier use	Full text not available Review articles (literature review, narrative review, scoping review, systematic review, meta-analysis) Editorials Letters to the Editor Conference Proceedings/Conference Abstracts Articles older than 2014 Articles not written in English Articles not focused on Pacifier use or dental complications

**Table 3:** Summary of studies.

Author (Year)	Age Range	Study Objective	Dental Complications	Prevalence of Complications	Factors Impacting Pacifier Use
M. J. Al Duliamy (2020) [24]	3 to 5 years	Assess the impact of two non-nutritive patterns on the development of anterior open bite in primary dentition and to compare which of these habits mostly affect open bite development	Anterior Open Bite	87% Children w/pacifier developed Open Bite	Lower Position of the Tongue
Alves et al. (2016) [25]	7 to 40 months	Verify the relationship between non-nutritive habits and malocclusions in children using day nurseries’ facilities	Open Bite, Posterior Crossbite	55% Children w/pacifier developed Open Bite	Intensity, duration, frequency
Amaral et al. (2017) [26]	24 to 36 months	Assess malocclusion in deciduous dentition and its association with prolonged breastfeeding, pacifier use, and perinatal health indicators pertaining to the periods immediately before and after birth.	Malocclusion (open bite, crossbite, overjet, canine relationship)	Children w/Pacifier 47.45% Open Bite 8.48% Crossbite 13.36% Left Canine Relationship 14.15% Right Canine Relationship 39.05% Overjet 62.33% Any Malocclusion	Duration
Arpalahti et al. (2024) [27]	0 to 7 years	Investigate the correlations between early childhood non-nutritive sucking habits and malocclusion. Specifically to test whether the use of a study pacifier has differing effects compared to other pacifiers and control, and whether the duration of pacifier use or digit sucking influence the occlusion.	Maxillary Arch Development	Children w/Pacifier 4% Posterior Crossbite w/study Pacifier 7% Posterior Crossbite w/other Pacifier	Duration, Frequency, Intensity
Assis et al. (2020) [28]	4 to 6 years	Analyze the prevalence and factors associated with malocclusions in preschool children.	Malocclusion, Anterior Open Bite	71.8% of children who used pacifiers had malocclusion	Unwanted Mechanical Forces, Frequency, Duration, Intensity
Cardoso et al. (2014) [29]	3 to 6 years	Investigate the associations between nutritive and non-nutritive sucking habits and the prevalence of anterior open bite, in children from Aragua-Venezuela and São Paulo-Brazil.	Anterior Open Bite	18.8% of children <3 years old w/pacifier had Anterior Open Bite 65.1% of children >3 years old w/pacifier had Anterior Open Bite	Duration
Chen et al. (2015) [30]	3 to 6 years	Assessed the effects of breast-feeding duration, bottle-feeding duration and non-nutritive sucking habits on the occlusal characteristics of primary dentition in 3–6-year-old children in Peking city.	Overjet, Impaired Lower Arch Development	10% of children using a pacifier after 1 year old had excessive overjet 30% had absence of lower arch development space	Duration
Costa et al. (2018) [31]	2 to 5 years	Evaluate the influence of breastfeeding and pacifier use on the occlusal status of preschool children.	Malocclusion	57.95% of children w/pacifier had severe malocclusion	Duration, Frequency, Intensity
Rosa et al. (2020) [32]	0 to 5 years	Aimed to investigate the association between preterm birth and primary-dentition malocclusion and how breastfeeding and the use of pacifiers are related to this association.	Malocclusion	60.6% of children w/pacifier had malocclusion	None Noted
Abreu Pegoraro et al. (2022) [33]	7 to 9 years	Evaluate the prevalence of malocclusion and its associated factors of children cared for by a PHC Service in Porto Alegre, Brazil.	Malocclusion, Anterior Open Bite	86.1% of children using pacifiers had malocclusion	None Noted
De Barros Miotto et al. (2015) [34]	3 to 5 years	Evaluate the prevalence of posterior cross bite and the possible association with deleterious oral habits in 3–5-year-old children from Vitória, Espírito Santo, Brazil.	Posterior Crossbite	49% of children w/pacifier use had posterior crossbite	None Noted
Lira et al. (2020) [35]	2 to 6 years	Evaluate the clinical behavior of sucking habits in children between 2 to 6 years old in a private (A1) and a public school (A2) in the state of Piauí.	Anterior Open Bite	75% of children who used a pacifier had anterior open bite	Duration, Frequency, Intensity
Sousa et al. (2014) [36]	3 to 5 years	Verify the prevalence of anterior open bite (AOB) and posterior crossbite (PC) in the primary dentition and the association with sociodemographic factors, presence and duration of nutritive and non-nutritive habits.	Malocclusion, Anterior Open Bite, Posterior Crossbite	87% of children who used a pacifier had anterior open bite (% increased in children using a pacifier > 36 months)	Duration
Feldens et al. (2016) [37]	2 to 5 years	Aim of the present study was to identify factors associated with the occurrence of distoclusion among preschool children in southern Brazil.	Distoclusion	34.5% of children w/past pacifier use had distoclusion 51.7% of children w/current pacifier use had distoclusion	None Noted
Feldens et al. (2023) [38]	0 to 12 months & 1 year	Investigate the long-term impact of breastfeeding and pacifier use during early childhood on increased overjet in adolescence.	Overjet	51.4% of children w/pacifier use had overjet	Unwanted Mechanical Forces
Freire et al. (2016) [39]	3 to 6 years	Evaluate the presence of non-nutritive sucking habits and their effects on the occlusion in the deciduous dentition in Spanish children.	Transverse Dimension	Children w/Pacifier Habit 14.6% Transversal Relationship 25% midline deviation 25.1% vertical relationship 41.5% sagittal relationship 60.5% any type of malocclusion	Duration
Galán-González et al. (2023) [40]	3 to 6 years	Assess posterior crossbite in deciduous dentition and its possible association to extrinsic factors.	Crossbite	35.5% of children w/pacifier use > 36 months had Posterior Crossbite 22.3% of children w/pacifier use < 36 months had Posterior Crossbite	Duration
Germa et al. (2016) [41]	3 years	Investigate risk factors specific to posterior crossbite and anterior open bite at the age of 3 years.	Posterior Crossbite, Anterior Open Bite	20% of children w/pacifier use had Posterior Crossbite 28% of children w/pacifier use had Anterior Open Bite	None Noted
Golovachova et al. (2021) [42]	3 to 5 years	Evaluate the prevalence of malocclusion and associated variables in the primary dentition among preschoolers in the city of Tbilisi.	Anterior Open Bite, Overjet, Class II Canine Relationship, Posterior Crossbite	In children who used a pacifier for less than 1.5 years: 22.5% Class II 12.2% Deep Overbite 9.2% Anterior Open Bite 8.9% Cross Bite Children with a prolonged pacifier sucking habit 27.9% Class II 10.9% Deep Overbite 13.7% Anterior Open Bite 12.2% Cross Bite	None Noted
Gomes et al. (2018) [43]	5 years	Evaluate association between psychological factors, socio-demographic conditions, oral habits and anterior open bite in five-year-old preschool children.	Anterior Open Bite	23.2% of children w/pacifier use had anterior open bite	None Noted
Herrera et al. (2022) [44]	5 years	Describe the frequency of high-arched palate and posterior crossbite at the age of 5 in children born preterm and to identify their respective factors.	High Arched Palate, Posterior Crossbite	Pacifier Sucking at 2 years 8.1% High-arched Palate 13.4% Posterior Crossbite	None Noted
Oliveira et al. (2021) [45]	18 to 72 months	Determine the prevalence of malocclusion and associated factors in children and the levels of knowledge of mothers participating in the child care group of a basic health unit.	Malocclusion, Anterior Open Bite	53.6% of children with pacifier use had malocclusion	Frequency, Duration
Lima et al. (2017) [46]	24 to 36 months 0 to 30 months	Investigate the effects of conventional and orthodontic pacifiers on the prevalence of malocclusion considering frequency, duration, and intensity of the sucking habit	Malocclusion, Anterior Open Bite, Overjet, Deep Overbite, Posterior Crossbite, Crowding		Frequency, Intensity, Duration
Moimaz et al. (2014) [47]	0 to 30 months	Assess the development of children under 30 months old by performing a longitudinal type study assessing sucking habits, nocturnal mouth breathing, and malocclusion.	Overjet, Overbite, Posterior Crossbite	Pacifier Sucking at 12 months - Overjet 22.8 - Overbite not effected - Open Bite 12.9 - Posterior Crossbite 0.7 18 months - Overjet 22.8 - Overbite not effected - Open Bite 12.9 - Posterior Crossbite 0.7 30 months - Overjet 26.9 - Overbite 9.3 - Open Bite 17.1 - Posterior Crossbite 1.7	None Noted
Moraes et al. (2021) [48]	2 to 5 years	Assess the direct and indirect pathways related to pacifier sucking habit and AOB in preschool children.	Anterior Open Bite	32.9% of pacifier users had anterior open bite at initial intervention	Duration
Nihi et al. (2015) [49]	2 to 4 years	Evaluate the association of pacifier-sucking habit with occlusal and oral myofunctional alterations in preschool children.	Malocclusion, Midline Deviation, Altered Canine Relation, Overjet, Overbite, Anterior Open Bite, Posterior Crossbite, Anterior Crossbite	Dental Developmental Problems with Pacifier use Malocclusion 77.8% Midline Deviation 36.1% Altered Canine Relation 52.8% Increased Overjet 38.9% Overbite 8.3% Anterior Open Bite 47.2% Posterior Crossbite 27.8% Anterior Crossbite 2.8%	Duration, Frequency
Pimenta et al. (2023) [50]	3 to 5 years	Study the prevalence of malocclusion in deciduous dentition and its associated factors.	Mesioclusion, Distoclusion, Posterior Crossbite, Crowding, Open Bite, Deep Overbite	With pacifier usage after 3 years old: Mesiocclusion: 3.7% Distoclusión: 63.0% Posterior Crossbite: 18.5% Crowding: 51.9% Open Bite: 51.9% Deep Overbite: 11.1%	Duration (after 3 years old)
Rai et al. (2022) [51]	3 to 7 years	Study the prevalence of oral habits among school going children with primary dentition, determine the association of oral habits with malocclusion in primary dentition, and compare the prevalence of oral habits based on gender, race, age, and grade	Malocclusion, Crowding, Molar Relationship, Canine Relationship, Midline Discrepancy, Corssbite, Open Bite, Overjet, Overbite	4.9% of the time pacifier use was associated with malocclusion	None Noted
Samohyl et al. (2017) [52]	0 to 25 years	Analyze selected malocclusion risk factors, their exposure time and overall malocclusion risk scores.	Malocclusion		Duration, Intensity
Scudine et al. (2021) [53]	4 years	Investigate the influence of pacifier removal on aspects of oro-dentofacial morphology and function in preschool children.	Maxillary and Mandibular Intercanine width, Maxillary and Mandibular intermolar width, Palatal Development, Overjet, Overbite		Duration
Silvestrini-Biavati et al. (2016) [54]	3 to 5 years	Evaluate the consequences of prolonged sucking habits on the development of the orofacial complex in deciduous dentition.	Anterior Open Bite	At 3 years old 19.5% of pacifier users had anterior open bite At 5 years old 8.7% of pacifier users had anterior open bite	None Noted
Traebert et al. (2021) [55]	6 years	Estimate the prevalence and factors associated with the anterior open bite in children in the first school year in a municipality in southern Brazil.	Anterior Open Bite	49.4% of children who used pacifiers had anterior open bite	None Noted
Traebert et al. (2020) [56]	6 years	Estimate the prevalence of malocclusions in the mixed dentition and to study possible association with practices of breastfeeding and suction habits among Brazilian schoolchildren.	Malocclusion, Overjet, Overbite, Posterior Crossbite, Anterior Open Bite, Anterior Crossbite	Dental Developmental Problems with Pacifier use 50.4% Class II or III Molar/Canine Relationship 36.3% Overjet 40.1% Overbite 17.4% Posterior Crossbite 18.1% Anterior Open Bite 8.1% Anterior Crossbite	None Noted
Wagner et al. (2015) [57]	0 to 3 years	Determine prevalence of malocclusion and associated risk factors in 3-year-old Thuringian children.	Malocclusion, Overjet, Overbite, Posterior Crossbite, Anterior Open Bite	50.5% of children who used a pacifier had malocclusion	None Noted
Zen et al. (2020) [14]	0 to 6 months	Evaluated the maxillary arch dimensions at birth and 6 months of life, and to verify the influence of pacifier use on palatal development.	Maxillary Arch Dimensions		Duration, Time

## References

[1] Pahel B.T., Rowan-Legg A., Quinonez R.B. (2018). A Developmental Approach to Pediatric Oral Health. Pediatr. Clin. N. Am..

[2] Elad D., Kozlovsky P., Blum O., Laine A.F., Po M.J., Botzer E., Dollberg S., Zelicovich M., Ben Sira L. (2014). Biomechanics of milk extraction during breast-feeding. Proc. Natl. Acad. Sci. USA.

[3] Sakalidis V.S., Kent J.C., Garbin C.P., Hepworth A.R., Hartmann P.E., Geddes D.T. (2013). Longitudinal Changes in Suck-Swallow-Breathe, Oxygen Saturation, and Heart Rate Patterns in Term Breastfeeding Infants. J. Hum. Lact..

[4] Fisher-Owens S.A., Gansky S.A., Platt L.J., Weintraub J.A., Soobader M.J., Bramlett M.D., Newacheck P.W. (2007). Influences on children’s oral health: A conceptual model. Pediatrics.

[5] American Academy of Pediatric Dentistry (2024). Policy on Pacifiers. In the Reference Manual of Pediatric Dentistry. https://www.aapd.org/globalassets/media/policies_guidelines/p_pacifiers.pdf.

[6] Jayavel N. (2023). Pacifiers: A Review. J. Indian Dent. Assoc. Tamilnadu (JIDAT).

[7] Pansy J., Zotter H., Sauseng W., Schneuber S., Lang U., Kerbl R. (2008). Pacifier use: What makes mothers change their mind?. Acta Paediatr..

[8] Bencosme J. (2016). Pacifiers for infants: What nurses need to know. Nursing.

[9] Sexton S., Natale R. (2009). Risks and benefits of pacifiers. Am. Fam. Physician.

[10] Nowak A.J., Warren J.J. (2000). Infant Oral Health and Oral Habits. Pediatr. Clin. N. Am..

[11] Larsson E. (1986). The effect of dummy-sucking on the occlusion: A review. Eur. J. Orthod..

[12] American Association of Orthodontists Pacifiers and Teeth: How Do Pacifiers & Thumb Sucking Impact Dental Development?. https://aaoinfo.org/whats-trending/can-pacifiers-and-thumb-sucking-affect-my-childs-teeth/?swpmtx=7f67245a558250a7e3d77bcbe9ef37e7&swpmtxnonce=239c641a82.

[13] De Carvalho F.M., Valadas L.A.R., Nogueira J.A.S., Lobo P.L.D., Pimentel F.L.D.S., da Silva Sacchetto M.S.L., de Alencar Júnior E.A., de Sousa A.P.R., de Oliveira Rodrigues S.G.S., De Aquino P.B. (2022). Breastfeeding, oral habits and malocclusions in the childhood: A literature review. J. Young Pharm..

[14] Zen I., Soares M., Pinto L., Ferelle A., Pessan J.P., Dezan-Garbelini C.C. (2020). Maxillary arch dimensions in the first 6 months of life and their relationship with pacifier use. Eur. Arch. Paediatr. Dent..

[15] Simões-Zenari M., Bitar M.L. (2010). Factors associated to bruxism in children from 4–6 years. Pro Fono.

[16] Medeiros R., Ximenes M., Massignan C., Flores-Mir C., Vieira R., Porporatti A.L., De Luca Canto G. (2018). Malocclusion prevention through the usage of an orthodontic pacifier compared to a conventional pacifier: A systematic review. Eur. Arch. Paediatr. Dent..

[17] Schmid K.M., Kugler R., Nalabothu P., Bosch C., Verna C. (2018). The effect of pacifier sucking on orofacial structures: A systematic literature review. Prog. Orthod..

[18] Tolppola O., Renko M., Sankilampi U., Kiviranta P., Hintikka L., Kuitunen I. (2022). Pacifier use and breastfeeding in term and preterm newborns—A systematic review and meta-analysis. Eur. J. Pediatr..

[19] Orovou E., Tzitiridou-Chatzopoulou M., Dagla M., Eskitzis P., Palaska E., Iliadou M., Iatrakis G., Antoniou E. (2022). Correlation between Pacifier Use in Preterm Neonates and Breastfeeding in Infancy: A Systematic Review. Children.

[20] Sadoun C., Templier L., Alloul L., Rossi C., Renovales I.D., Sanchez I.N., Sahagun P.M. (2024). Effects of non-nutritive sucking habits on malocclusions: A systematic review. J. Clin. Pediatr. Dent..

[21] Maloney B., Leith R. (2023). An Update in Non-Nutritive Sucking Habit Cessation. J. Ir. Dent. Assoc..

[22] Duncan K., McNamara C., Ireland A.J., Sandy J.R. (2008). Sucking habits in childhood and the effects on the primary dentition: Findings of the Avon Longitudinal Study of Pregnancy and Childhood. Int. J. Paediatr. Dent..

[23] Tricco A.C., Lillie E., Zarin W., O’Brien K.K., Colquhoun H., Levac D., Moher D., Peters M.D.J., Horsley T., Weeks L. (2018). PRISMA Extension for Scoping Reviews (PRISMA-ScR): Checklist and Explanation. Ann. Intern. Med..

[24] Al Duliamy M.J. (2020). Impact of two non-nutritive sucking patterns on the development of anterior open bite in children of two kindergartens in Baghdad city. J. Baghdad Coll. Dent..

[25] Alves F.B., Wambier D.S., Alvarez J.H., da Rocha J.C., Kummer T.R., de Castro V.C., Cabral H., Kozlowski V.A. (2016). Children using Day Nurseries’ Facilities can be Associated with more Risk to Nonnutritive Sucking Habits. J. Contemp. Dent. Pract..

[26] Amaral C.C., da Costa V.P.P., Azevedo M.S., Pinheiro R.T., Demarco F.F., Goettems M.L. (2017). Perinatal health and malocclusions in preschool children: Findings from a cohort of adolescent mothers in Southern Brazil. Am. J. Orthod. Dentofac. Orthop..

[27] Arpalahti I., Hanninen K., Tolvanen M., Varrela J., Rice D.P. (2024). The effect of early childhood non-nutritive sucking behavior including pacifiers on malocclusion: A randomized controlled trial. Eur. J. Orthod..

[28] Assis W.C., Pereira J.S., Silva Y.S., Brito F.R., Nunes L.A., Ribeiro Í.J.S., Casotti C.A. (2020). Factors Associated with Malocclusion in Preschool Children in a Brazilian Small Town. Pesqui. Bras. Odontopediatr. Clín. Integr..

[29] Cardoso A.C., Bello M.G.D., Vellini-Ferreira F., Ferreira-Santos R.I. (2014). Sucking habits and anterior open bite among Venezuelan and Brazilian children. Braz. J. Oral Sci..

[30] Chen X., Xia B., Ge L. (2015). Effects of breast-feeding duration, bottle-feeding duration and non-nutritive sucking habits on the occlusal characteristics of primary dentition. BMC Pediatr..

[31] Costa C.T.D., Shqair A.Q., Azevedo M.S., Goettems M.L., Bonow M.L.M., Romano A.R. (2018). Pacifier use modifies the association between breastfeeding and malocclusion: A cross-sectional study. Braz. Oral. Res..

[32] da Rosa D.P., Bonow M.L.M., Goettems M.L., Demarco F.F., Santos I.S., Matijasevich A., Barros A.J., Peres K.G. (2020). The influence of breastfeeding and pacifier use on the association between preterm birth and primary-dentition malocclusion: A population-based birth cohort study. Am. J. Orthod. Dentofac. Orthop..

[33] Pegoraro N.A., Santos C.M.D., Colvara B.C., Rech R.S., Faustino-Silva D.D., Hugo F.N., Hilgert J.B. (2021). Prevalence of malocclusion in early childhood and its associated factors in a primary care service in Brazil. Codas.

[34] Miotto M.H.M.D.B., Cavalcante W.S., Godoy L.M., Campos D.M., Barcellos L.A. (2015). Prevalence of Posterior Cross Bite in 3-5-Year-Old Children from Vitória, Brazil. Pesqui. Bras. Odontopediatr. Clín. Integr..

[35] Lira A.d.L.S.d., Santos A.R. (2020). Influence of non-nutritive sucking habits on anterior open bite. Braz. J. Oral Sci..

[36] Sousa R.V.D., Ribeiro G.L.A., Firmino R.T., Martins C.C., Granville-Garcia A.F., Paiva S.M. (2014). Prevalence and Associated Factors for the Development of Anterior Open Bite and Posterior Crossbite in the Primary Dentition. Braz. Dent. J..

[37] Feldens C.A., Martins R.P., Maciel R.R., Vargas-Ferreira F., Kramer P.F. (2016). Factors Associated with the Accurrence of Distoclusion in the Primary Dentition: A Hierarchical Analysis. J. Clin. Pediatr. Dent..

[38] Feldens C.A., Petracco L.B., Nascimento G.G., Li H., Vitolo M.R., Peres K.G. (2023). Breastfeeding Protects from Overjet in Adolescence by Reducing Pacifier Use: A Birth Cohort Study. Nutrients.

[39] Freire G.L., de Deza J.E.S., da Silva I.R., Oliveira L.B., Torrent J.U., Quesada J.B. (2016). Non-nutritive sucking habits and their effects on the occlusion in the deciduous dentition in children. Eur. J. Paediatr. Dent..

[40] Galan-Gonzalez A.F., Dominguez-Reyes A., Cabrera-Dominguez M.E. (2023). Influence of bad oral habits upon the development of posterior crossbite in a preschool population. BMC Oral Health.

[41] Germa A., Clement C., Weissenbach M., Heude B., Forhan A., Martin-Marchand L., Bonet M., Vital S., Kaminski M., Nabet C. (2016). Early risk factors for posterior crossbite and anterior open bite in the primary dentition. Angle Orthod..

[42] Golovachova E., Mikadze T., Darjania O. (2021). Prevalence of Malocclusion and Associated Variables in Preschool Children of Tbilisi, Georgia. Open Dent. J..

[43] Gomes M.C., Neves E.T.B., Perazzo M.F., Martins C.C., Paiva S.M., Granville-Garcia A.F. (2018). Association between psychological factors, socio-demographic conditions, oral habits and anterior open bite in five-year-old children. Acta Odontol. Scand..

[44] Herrera S., Pierrat V., Kaminski M., Benhammou V., Marchand-Martin L., Morgan A.S., Le Norcy E., Ancel P.-Y., Germa A. (2022). Risk Factors for High-Arched Palate and Posterior Crossbite at the Age of 5 in Children Born Very Preterm: EPIPAGE-2 Cohort Study. Front. Pediatr..

[45] Oliveira A.C.J., Paula T.M.d., Maschio D.F., Jaccottet C.M., Salas M.M.S., Lund R.G. (2021). Malocclusion and Associated Factors in Early Childhood and Knowledge Level of Mothers from Childcare Groups. Pesqui. Bras. Odontopediatr. Clín. Integr..

[46] Lima A.A., Alves C.M., Ribeiro C.C., Pereira A.L., da Silva A.A., Silva L.F., Thomaz E.B. (2017). Effects of conventional and orthodontic pacifiers on the dental occlusion of children aged 24-36 months old. Int. J. Paediatr. Dent..

[47] Moimaz S.A.S., Garbin A.J.Í., Lima A.M.C., Lolli L.F., Saliba O., Garbin C.A.A.S. (2014). Longitudinal study of habits leading to malocclusion development in childhood. BMC Oral Health.

[48] Moraes R.B., Knorst J.K., Pfeifer A.B.R., Vargas-Ferreira F., Ardenghi T.M. (2021). Pathways to anterior open bite after changing of pacifier sucking habit in preschool children: A cohort study. Int. J. Paediatr. Dent..

[49] Nihi V.S., Maciel S.M., Jarrus M.E., Nihi F.M., Salles C.L., Pascotto R.C., Fujimaki M. (2015). Pacifier-sucking habit duration and frequency on occlusal and myofunctional alterations in preschool children. Braz. Oral Res..

[50] Pimenta C., Esperancinha C., Bernardo M., Mendes S. (2023). Malocclusion in primary dentition: A cross-sectional study in a Lisbon population. Rev. Port. Estomatol. Med. Dentária Cir Maxilofac..

[51] Rai A., Koirala B., Dali M., Shrestha S., Shrestha A., Niraula S.R. (2022). Prevalence of Oral Habits and its Association with Malocclusion in Primary Dentition among School Going Children of Nepal. J. Clin. Pediatr. Dent..

[52] Samohyl M., Nadazdyova A., Hirjak M., Hirosova K., Vondrova D., Argalasova L., Jurkovicova J. (2017). The Analysis of Selected Malocclusion Risk Factors: A Pilot Study. Pesqui. Bras. Odontopediatr. Clín. Integr..

[53] Scudine K.G.D.O., Freitas C.N.D., Nascimento De Moraes K.S.G., Bommarito S., Possobon R.D.F., Boni R.C., Castelo P.M. (2021). Multidisciplinary Evaluation of Pacifier Removal on Oro-Dentofacial Structures: A Controlled Clinical Trial. Front. Pediatr..

[54] Silvestrini-Biavati A., Salamone S., Silvestrini-Biavati F., Agostino P., Ugolini A. (2016). Anterior open-bite and sucking habits in Italian preschool children. Eur. J. Paediatr. Dent..

[55] Traebert E., Marcos V.F., Willig D.Q., Traebert J. (2021). Prevalence of anterior open bite and associated factors in schoolchildren in a municipality of southern Brazil. Rev. Odontol. UNESP.

[56] Traebert E., Zanini F.A., Nunes R.D., Traebert J. (2020). Nutritional and non-nutritional habits and occurrence of malocclusions in the mixed dentition. Acad. Bras. Cienc..

[57] Wagner Y., Heinrich-Weltzien R. (2015). Occlusal characteristics in 3-year-old children--results of a birth cohort study. BMC Oral Health.

[58] Melink S., Vagner M.V., Hocevar-Boltezar I., Ovsenik M. (2010). Posterior crossbite in the deciduous dentition period, its relation with sucking habits, irregular orofacial functions, and otolaryngological findings. Am. J. Orthod. Dentofac. Orthop..

[59] Warren J.J., Bishara S.E. (2002). Duration of nutritive and nonnutritive sucking behaviors and their effects on the dental arches in the primary dentition. Am. J. Orthod. Dentofac. Orthop..

[60] Frequently Asked Questions (FAQ) American Academy of Pediatric Dentistry. https://www.aapd.org/resources/parent/faq/.

[61] Poyak J. (2006). Effects of pacifiers on early oral development. Int. J. Orthod. Milwaukee.

[62] Christensen J.R., Fields H.W., Adair S.M., Pinkham J., Casamassimo P., Fields H.W., McTigue D.J., Nowak A.J. (2005). Chapter 6 Oral Habits. Pediatric Dentistry: Infancy through Adolescence.

[63] Lone I.M., Zohud O., Midlej K., Paddenberg E., Krohn S., Kirschneck C., Proff P., Watted N., Iraqi F.A. (2023). Anterior Open Bite Malocclusion: From Clinical Treatment Strategies towards the Dissection of the Genetic Bases of the Disease Using Human and Collaborative Cross Mice Cohorts. J. Pers. Med..

[64] Peres K.G., Peres M.A., Thomson W.M., Broadbent J., Hallal P.C., Menezes A.B. (2015). Deciduous-dentition malocclusion predicts orthodontic treatment needs later: Findings from a population-based birth cohort study. Am. J. Orthod. Dentofac. Orthop..

[65] Masood Y., Masood M., Zainul N.N.B., Araby N.B.A.A., Hussain S.F., Newton T. (2013). Impact of malocclusion on oral health related quality of life in young people. Health Qual. Life Outcomes.

[66] Zhang M., McGrath C., Hägg U. (2006). The impact of malocclusion and its treatment on quality of life: A literature review. Int. J. Paediatr. Dent..

[67] Assaf D.D.C., Knorst J.K., Busanello-Stella A.R., Ferrazzo V.A., Berwig L.C., Ardenghi T.M., Marquezan M. (2021). Association between malocclusion, tongue position and speech distortion in mixed-dentition schoolchildren: An epidemiological study. J. Appl. Oral. Sci..

[68] Leavy K.M., Cisneros G.J., Leblanc E.M. (2016). Malocclusion and its relationship to speech sound production: Redefining the effect of malocclusal traits on sound production. Am. J. Orthod. Dentofac. Orthop..

[69] Lindsay G., Dockrell J., Letchford B., Mackie C. (2002). Self esteem of children with specific speech and language difficulties. Child. Lang. Teach. Ther..

[70] Strutt C., Khattab G., Willoughby J. (2021). Does the duration and frequency of dummy (pacifier) use affect the development of speech?. Int. J. Lang. Commun. Disord..

[71] Magalhães I.B., Pereira L.J., Marques L.S., Gameiro G.H. (2010). The influence of malocclusion on masticatory performance: A systematic review. Angle Orthod..

[72] Maharani D.A., Adiatman M., Rahardjo A., Burnside G., Pine C. (2017). An assessment of the impacts of child oral health in Indonesia and associations with self-esteem, school performance and perceived employability. BMC Oral Health.

[73] Agou S., Locker D., Streiner D.L., Tompson B. (2008). Impact of self-esteem on the oral-health-related quality of life of children with malocclusion. Am. J. Orthod. Dentofac. Orthop..

[74] Martins-Júnior P., Marques L., Ramos-Jorge M.L. (2012). Malocclusion: Social, functional and emotional influence on children. J. Clin. Pediatr. Dent..

[75] Degan V.V., Puppin-Rontani R.M. (2004). Prevalence of pacifier-sucking habits and successful methods to eliminate them--a preliminary study. J. Dent. Child..

